# Stress level in adolescents wearing corrective brace for idiopathic scoliosis: study with BSSQ questionnaires

**DOI:** 10.1186/1748-7161-10-S1-P32

**Published:** 2015-01-19

**Authors:** Edyta Kinel, Anna Podolska-Piechocka, Magdalena Sobieska, Tomasz Kotwicki

**Affiliations:** 1 Department of Rheumatology and Rehabilitation, University of Medical Sciences, Poznan, Poland; 2 Department of Paediatric Orthopaedics and Traumatology, University of Medical Sciences, Poznan, Poland

## Objective

Evaluation of the stress level in adolescents with idiopathic scoliosis (IS) according to: age, clinical deformity, radiological deformity, hours in brace per day and place of residence.

## Material and methods

The study involved 80 adolescents (71 girls and 9 boys), fulfilling the inclusion criteria: age range between 11.0 and 16.0 years, Cobb angle range between 20 and 45 degrees, wearing Chêneau orthosis for more than 3 months, at least 12 hours per day. The group was analyzed in subgroups according to (1) age: 11.0-13.0 years (n=40) versus 14.0- 16.0 years (n=40), (2) brace wearing time: less than 20h (n=45) versus more than 20h per day (n=35), (3) place of residence: village (n= 34), town (n= 20) or city (n= 26). Maximal angle of trunk rotation (ATR max) was measured using Bunnell scoliometer.

Bad Sobernheim Stress Questionnaires (BSSQ), both the Brace and the Deformity versions were used, the former estimates the stress the patients have whilst wearing brace, the latter estimates the stress induced with body deformation.

## Results

The age of the group was 13.4 ± 1.6 years. Cobb angle was 31.1 ± 8.1 degrees. The ATR max was 8.8 ± 3.7 degrees. Total brace wearing time was 16.3 ± 11.0 months and daily wearing time was 17.2 ± 4.3 hours. The BSSQ median was 13 for the BSSQ Deformity and 12 for the BSSQ Brace (moderate stress level). There was no statistically significant difference between subgroups according to age. There was statistically significant weak negative correlation between Cobb angle and the BSSQ Brace score, r= -0.238, p= 0.033. There were statistically significant weak negative correlations between the ATR max and either BSSQ Deformity (r= -0.270, p= 0.015) or BSSQ Brace scores (r= -0.267, p= 0.016). The higher Cobb angle and ATR max, the higher was the stress level. Adolescents wearing the brace for more than 20h per day presented slightly higher stress level (median BSSQ = 13 and 12 for BSSQ Deformity and Brace, respectively) comparing to those wearing for less than 20h (median BSSQ 15 and 12 for BSSQ Deformity and Brace, respectively). There was no significant difference of the stress level related to the place of residence.

## Conclusions

Patients with more clinical (ATR) or radiological (Cobb) deformity experienced more stress. Conservative treatment did not severely impact the level of stress of the adolescent patients with IS wearing Chêneau orthosis comparing to stress induced be the deformity itself.

